# Responsible use of generative AI in Chinese vocational education: eye-tracking evidence on source verification and immediate near-transfer across three information-seeking conditions

**DOI:** 10.3389/fpsyg.2026.1946196

**Published:** 2026-09-30

**Authors:** Ji Ma, Hongjie Yue, Xinyang Li

**Affiliations:** 1 School of Marxism, Liaoning Economy Vocational and Technical College, Shenyang, China; 2 School of Design, East China Normal University, Shanghai, China

**Keywords:** eye tracking, generative artificial intelligence, immediate near transfer, information seeking, responsible AI, source verification

## Abstract

**Introduction:**

Generative artificial intelligence (GenAI) can streamline information screening and integration in vocational learning, but easy access to fluent answers may reduce students’ tendency to check sources. Drawing on a dual-process perspective, this study compared GenAI-only, conventional web-search, and hybrid-search conditions in terms of source verification, task trust, perceived cognitive load, and condition-supported immediate near-transfer performance.

**Methods:**

Ninety-six vocational college students took part in a three-group, between-subjects eye-tracking experiment. After stratification by year of study, disciplinary cluster, and prior AI-use experience, they were randomly assigned to the three conditions (*n* = 32 each). Eye-tracking data, screen-recorded behavioral logs, questionnaires, and task scores were examined using between-group comparisons, correlation analyses, and hierarchical regression.

**Results:**

GenAI-only participants completed the two timed tasks fastest and reported the lowest perceived cognitive load and the highest task trust, but they devoted less attention to source evidence, made fewer cross-verification attempts, and revised their answers less often. Conventional web search involved more multi-source browsing and evidence comparison but required the most time. Hybrid search showed the highest source-evidence dwell proportion (27.9%), cross-verification-attempt rate (78.1%), and immediate near-transfer score (86.99), and outperformed both other conditions on near-transfer performance. After adjustment for search condition and prior tool experience, source-evidence dwell proportion (*β* = 0.28, *p* = 0.006) and cross-verification attempts (*β* = 0.31, *p* = 0.002) remained positively associated with near-transfer performance, whereas task trust did not.

**Conclusion:**

The three search modes showed different trade-offs between efficiency, verification, and task performance. GenAI-only search reduced completion time and overall perceived cognitive load, whereas hybrid search was associated with more source checking and better immediate near-transfer performance under the assigned condition. These findings suggest that GenAI can support problem framing and information organization when learners continue to verify evidence and retain responsibility for final judgments.

## 1 Introduction

Against the backdrop of accelerating digital transformation and rapid advances in artificial intelligence, generative artificial intelligence (GenAI) is reshaping how knowledge is acquired, how learning support is delivered, and how competencies are developed in education (Kasneci et al., 2023; Yan et al., 2024). Compared with conventional web search, GenAI-assisted search can reduce the operational effort associated with iterative searching or query reformulation, result screening, multi-page navigation, and information integration by providing synthesized, task-oriented responses (Kaiser et al., 2025; Cai and Tian, 2025). It can generate structured responses at relatively low cost and help learners rapidly develop a problem representation and an initial solution (Kasneci et al., 2023). At the same time, it may compress the multi-source comparison and evidence-verification processes through which learning occurs, increasing the risks of cognitive dependence, acceptance of inaccurate information, and weakened critical judgment (Lee et al., 2025). United Nations Sustainable Development Goal 4 calls for inclusive and equitable quality education and lifelong learning opportunities that support sustainable individual and social development (OECD, 2026). From the perspective of sustainable vocational education, source verification and learning transfer are essential components of high-quality digital literacy. This is particularly important in occupational tasks involving regulations, safety, procedural standards, time-sensitive information, or other high-consequence decisions. In such tasks, the reliability and scope of a source directly affect the quality of professional judgment. Learning transfer, in turn, indicates whether students can apply reliable information and verification strategies in new vocational contexts. Together, these competencies support the effective learning outcomes, occupationally relevant skills, and lifelong-learning capacity emphasized in SDG 4.

Accordingly, the educational value of AI tools depends closely on how students trust them and how they verify their outputs (Fu and Weng, 2024). Conventional web search prompts learners to enter multiple webpages, compare sources, and integrate information, but at a greater cost in time and cognitive effort. Hybrid search may preserve advantages of both modes by allowing learners to move flexibly among AI generation, keyword expansion, web search, source verification, and answer revision as the task requires. This combination may offer a workable balance between operational cost and evidential judgment (Boetje et al., 2026; Yen et al., 2024). Different search modes are consequently more than alternative tools; they may elicit different information-processing pathways, forms of trust, and transfer outcomes.

Tool-level complementarity, however, does not guarantee that learners will voluntarily incur the cost of verification. When learners have limited domain knowledge, difficulty recognizing errors, or little perceived benefit from additional checking, they may conserve cognitive resources and prefer to accept fluent, immediately available answers. Research on technology adoption likewise indicates that effort expectancy, social influence, and facilitating conditions shape students’ intentions to use GenAI (BinJwair, 2025). Research on hybrid search must distinguish effectiveness under an assigned verification requirement from learners’ spontaneous adoption in naturalistic settings.

In authentic learning contexts, this issue may become a risk of overreliance. The central question is not simply whether AI and search tools are both available. It is also when learners are prompted to verify, whether verification becomes part of the actual response process, and whether tool design preserves their cognitive engagement with evidence and final judgment.

Several gaps remain in the literature. First, most research has focused on general higher education or broad learning contexts, with comparatively little attention to vocational education, where practical application and skill transfer are central (Sun and Tian, 2026). Second, technology acceptance, learning outcomes, and user attitudes have often been examined separately, leaving the process links among search mode, task trust, source verification, and learning transfer insufficiently explained (García-Alonso et al., 2024; Chen et al., 2025). In learning environments where GenAI and conventional search coexist, prior work has also not adequately distinguished tool affordances from learners’ actual verification behavior. Although hybrid search may combine generative efficiency with external evidence checking, direct evidence remains limited on whether learners will voluntarily invest additional cognitive resources in verification. It is also unclear whether advantages observed under experimental requirements will translate into spontaneous use in natural learning settings.

Against this background, the present study examined vocational college students using a multimodal design that combined eye tracking, behavioral logs, questionnaires, and task scores. Under a common, explicit verification requirement, participants were randomly assigned to one of three information-seeking conditions. Responsible GenAI use served as a process-based interpretive framework for explaining how GenAI-only search, conventional web search, and hybrid search affected source-related attention, verification behavior, overall perceived cognitive load, and immediate near-transfer performance. The study addressed three research questions.

*RQ1*: How do the three information-seeking conditions differ in source related visual attention and observable verification behavior?

*RQ2*: How do the three search conditions differ in task trust, overall perceived cognitive load, and condition-supported immediate near-transfer performance?

*RQ3*: To what extent are attention to source evidence and cross source verification associated with immediate near transfer performance after controlling for search condition, prior AI use experience, and prior web search experience?

By answering these questions, this study extends research on GenAI-supported learning beyond efficiency outcomes toward a process-based account of trust, verification, and transfer. These findings also inform the responsible integration of AI in vocational education. Such integration should help students move from merely using AI efficiently to using it responsibly while supporting high-quality, accountable, and sustainable vocational education.

## 2 Literature review

### 2.1 The educational value and risks of generative AI and the digital-literacy requirements it creates

Research on GenAI-supported learning has gradually shifted from demonstrating tool effectiveness to explaining learning mechanisms (Kasneci et al., 2023). Early studies primarily emphasized efficiency gains in question answering, writing support, feedback, and resource organization (Kasneci et al., 2023; Cai and Tian, 2025). Because these tools are easy to access, highly interactive, and responsive, they can reshape learning support and create new opportunities for personalized learning, collaborative learning, and just-in-time tutoring. GenAI has consequently been framed as an important driver of educational digital transformation and incorporated into discussions of quality education and sustainable learning capacity (OECD, 2026; Miao and Holmes, 2023). More recent work has extended its educational value beyond efficiency to include changes in motivation, task understanding, and knowledge construction (Yan et al., 2024). AI-generated content may improve comprehensibility, accessibility, and contextualized support, helping students organize a problem, develop preliminary ideas, search the literature, define questions, and identify possible lines of inquiry.

The educational value of GenAI, however, cannot be judged solely by the speed with which it produces answers. AI outputs are often fluent, well structured, and immediate. Low-quality AI-generated content produced without sufficient human oversight—referred to here as AI-generated “slop”—may contain factual errors, missing context, or opaque sourcing. Its polished appearance may make it more difficult for learners to distinguish readability from reliability (Kasneci et al., 2023; Lee et al., 2025), encouraging them to equate expressive quality with evidential quality and reducing their motivation to verify (Anderl et al., 2024; Martín-Moncunill and Alonso Martínez, 2025). Research on tools such as ChatGPT has identified both educational opportunities and risks, including personalized feedback and learning support alongside concerns about academic integrity, weakened critical thinking, and difficulty evaluating generated information. GenAI-supported learning may thus improve efficiency while also encouraging cognitive offloading, overtrust, and source neglect.

From a digital-literacy perspective, the key issue is no longer simply whether students can operate an AI tool, but whether they can preserve their capacity for judgment, verification, and transfer while using it. For vocational education, digital literacy should extend beyond prompt construction and basic tool use to include source sensitivity, evidence verification, bias recognition, metacognitive monitoring, and responsible use (Bråten et al., 2011; Walraven et al., 2009; Salmerón et al., 2020). Verification requirements should also be calibrated to task risk. High-consequence tasks involving regulation, workplace safety, food safety, consumer rights, or financial judgment require careful checking of source provenance, scope, and currency. Low-risk, open-ended, or creative tasks do not necessarily warrant the same verification intensity (OECD, 2026; Miao and Holmes, 2023).

Existing research has established both the learning-support value and the risks of GenAI, but has paid insufficient attention to how vocational students evaluate sources, calibrate trust, and transfer what they have learned in authentic tasks. This gap makes the information-seeking environment itself a key part of the problem: different search-support conditions may alter how learners distribute effort between rapid answer construction and source verification. The next question is therefore whether GenAI-only, conventional web, and hybrid search produce distinct patterns of trust, verification, cognitive demand, and transfer.

### 2.2 Trust, information verification, and learning transfer across search-support conditions

Search-support conditions influence how students obtain, evaluate, and use information. Conventional web search requires learners to formulate keywords, inspect search results, enter webpages, and compare sources. This process generally demands more time and cognitive effort, but it also creates opportunities to encounter diverse information, discriminate among sources, and reach more considered judgments. AI-assisted retrieval, by contrast, generates integrated responses conversationally, reducing the demands of result screening and information organization and allowing learners to move more quickly to answer construction. The same convenience, however, may compress browsing, comparison, and verification and thereby increase reliance on ready-made answers.

Task trust provides an important lens for understanding how search modes influence learning. An appropriate level of trust enables students to accept tool support and proceed with a task. Conventional search may not produce stronger subjective trust, but it requires learners to build a basis for judgment through source clicks, webpage visits, and comparisons across pages. Hybrid search offers an intermediate pathway. Students can use AI to clarify a problem, generate keywords, or develop an initial answer, and then use a search engine to confirm facts, verify sources, and revise the response.

The availability of hybrid search does not mean that learners will spontaneously adopt verification strategies. Learners with limited domain knowledge may be drawn to the fluency and immediacy of generated answers, remain on a low-cost information pathway, or fail to recognize errors and uncertainty that would trigger additional checking. The educational value of hybrid search depends on more than the availability of both tools. It also depends on whether task requirements, verification prompts, and assessment criteria activate source awareness and direct additional cognitive effort toward critical facts and evidence.

From a learning-transfer perspective, the effects of search mode cannot be evaluated by completion speed alone. Tool-supported performance must also be distinguished from durable capability development: performance gains produced by GenAI do not necessarily constitute learning or skill acquisition (Fauth and González-Martínez, 2021; Melumad and Yun, 2025; Yan et al., 2025). Transfer requires students to understand the grounds and rules underlying information and to reapply that understanding in a new task. Reliance on ready-made AI answers may improve immediate efficiency without producing deeper understanding. This study examines whether learners can reapply source verification, evidence comparison, and answer-revision strategies to a structurally similar task while retaining their assigned support condition. This performance is not equated with long-term capability development.

Although prior studies have separately established the importance of AI-assisted retrieval, conventional search, task trust, and learning transfer, direct comparisons among the three retrieval modes remain limited. In particular, few studies have compared GenAI-only, conventional web, and hybrid search within the same task framework. Comparing these conditions can establish whether outcomes differ, but outcome differences alone cannot show how learners allocate attention to source evidence during the search process. Process-level measures are therefore needed to determine whether differences in trust and transfer are accompanied by differences in source-oriented attention and verification.

### 2.3 Eye tracking in research on information verification and learning processes

Source verification is both an information-evaluation behavior and a process of attentional allocation. Questionnaires and final scores alone cannot establish whether students attended to source cues or repeatedly moved among answers, evidence, and response areas. Eye tracking records visual activity during a task, including fixation duration, fixation count, AOI dwell proportion, revisit count, and transitions across regions. These indices can reveal how attention is distributed during information search, evidential judgment, and answer construction and thereby provide process-level evidence relevant to source verification. These affordances have made eye tracking particularly useful for studying source evaluation in digital environments (Gottschling et al., 2019; Liu and Cui, 2025; Hessels et al., 2025).

Research in educational technology, digital reading, and interface evaluation has shown that eye tracking is well suited to the analysis of cognitive processing in complex digital environments. Studies commonly define AOIs and examine how gaze is distributed and shifts across information regions to characterize attentional priorities, depth of processing, and task strategies. Dwell time in source regions, revisits, and cross-region transitions are particularly relevant to information verification. Learners with stronger digital literacy are more likely to inspect authorship, provenance, publication date, citation cues, and the location of supporting evidence rather than attending only to main content. Conversely, attention concentrated on answer or content areas, with source cues largely neglected, may indicate limited source awareness and shallower evidential judgment. This interpretation follows prior work on online source evaluation and gaze-based indicators of credibility assessment (Gottschling and Kammerer, 2021; Guan and Lin, 2025; Tsai et al., 2022).

When GenAI enters the learning environment, information seeking is no longer a linear sequence of webpage browsing. AI answer generation, inspection of search results, source evaluation, and answer revision may occur in parallel or in repeated cycles. Eye tracking can reveal whether learners remain primarily within the AI-response region, redirect attention to source evidence, or move repeatedly among generated answers, webpage content, search results, and the response area. When combined with screen recordings, behavioral logs, questionnaires, and task scores, it can support an integrated account of visual attention, overt behavior, subjective experience, and learning outcomes across search conditions. Multimodal learning analytics can strengthen this inference by triangulating gaze with recorded action and performance evidence (Järvelä et al., 2021; Mohammadi et al., 2025).

Although eye-tracking research has illuminated attentional allocation during digital reading and information evaluation, multimodal process evidence remains limited in GenAI-supported learning. Integrating visual-attention indices, behavioral logs, subjective measures, and transfer-task scores can reveal where attention is allocated and whether verification actions occur. These measures, however, do not by themselves explain why fluent AI output may encourage rapid acceptance or why verification prompts may trigger more deliberate checking. Interpreting such process differences therefore requires an explanatory account of how heuristic and analytic processing are engaged.

### 2.4 Theoretical framework: AI reliance and verification from a dual-process perspective

To provide this explanatory account, the study uses a dual-process, heuristic–analytic perspective as its primary framework. Dual-process accounts propose that fast, automatic Type 1 processing typically generates a default response, whereas working-memory-dependent Type 2 processing becomes more fully engaged when more deliberate judgment is required (Evans and Stanovich, 2013). In AI-assisted decision making, Buçinca et al. (2021) further showed that users may rely on a generalized heuristic trust in AI rather than analytically evaluating each recommendation. Cognitive forcing functions can reduce such overreliance, although they also increase perceived effort.

From this perspective, the speed, fluency, and integrated presentation of GenAI output can reduce operational friction while increasing heuristic trust and reducing spontaneous verification (Lee and See, 2004; Vasconcelos et al., 2023). External evidence, verification prompts, and the affordances of hybrid search may instead introduce limited, purposeful “cognitive friction” at critical claims, prompting learners to compare sources, revise answers, and engage in more analytic processing. The objective is not to maximize cognitive load, but to shift cognitive effort away from mechanical search operations and toward necessary evidential judgment. Trust calibration and responsible human–AI interaction serve as complementary concepts for explaining whether learners adjust trust in response to evidence and retain responsibility for final judgment. They are not treated as additional independent theoretical systems.

Within this framework, information-seeking condition is the experimental manipulation. Attention to the source-evidence AOI, cross-verification attempts, and answer revision are key process indicators. Task trust and overall perceived cognitive load characterize subjective states, whereas condition-supported immediate near-transfer performance is a principal learning outcome.

## 3 Methodology

This study employed a three condition between subjects experimental design and integrated multimodal data from eye tracking, screen recordings, behavioral logs, questionnaires, and task scores. After data cleaning and reliability checks, descriptive statistics, between-group tests, correlation analyses, and regression analyses were used to examine source-related attention, verification behavior, task trust, overall perceived cognitive load, and immediate near-transfer performance across information-seeking conditions. Responsible GenAI use served as a process-based interpretive framework rather than a measured composite variable. It was interpreted through task trust considered alongside attention to sources, evidence verification, answer revision, and condition supported immediate near-transfer performance. The research pathway is shown in Figure 1.

**Figure 1:**
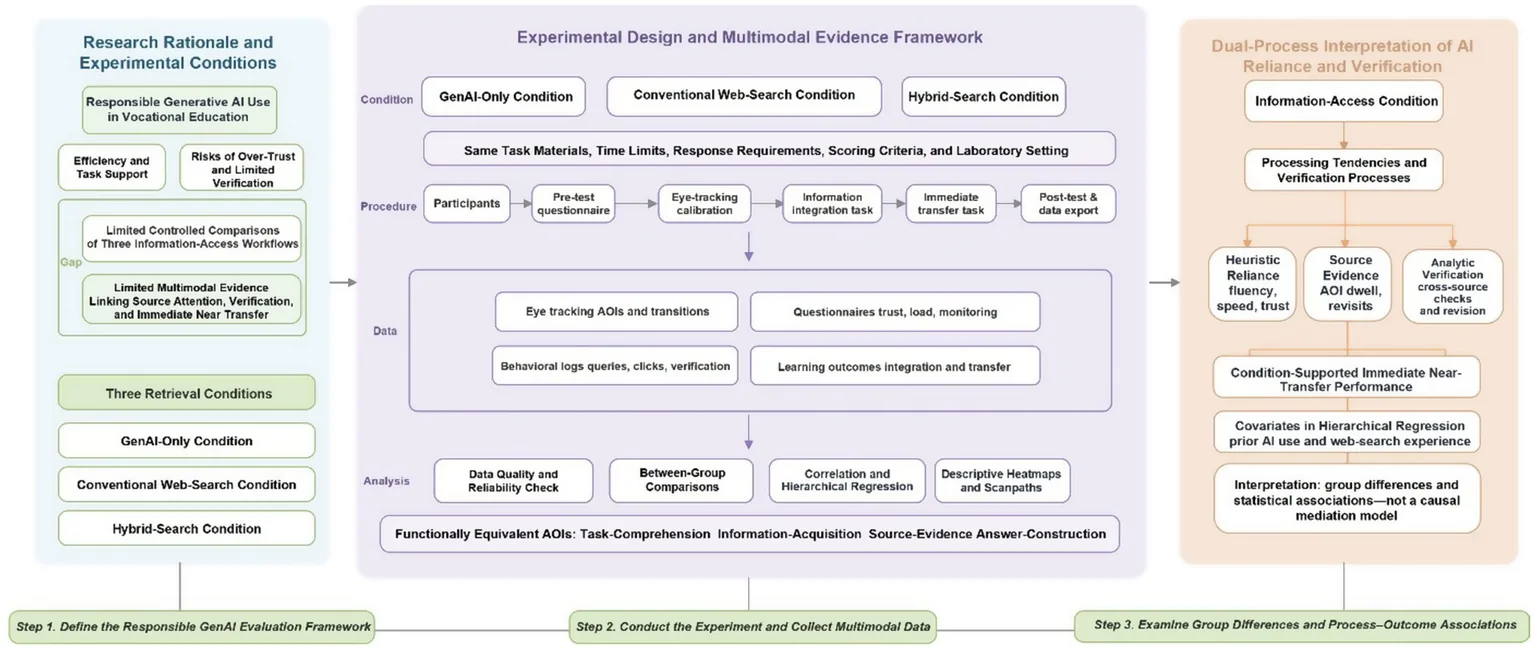
Research pathway and analytical framework for evaluating responsible GenAI use.

### 3.1 Research design and participants

#### 3.1.1 Study design, recruitment, and sample size

This study employed a three condition between subjects controlled experiment involving students enrolled at a vocational college in China. Participants were recruited between 9 May and 9 June 2026, yielding a convenience sample. They were 18–25 years of age and represented vocational fields including electromechanical engineering and manufacturing, finance and commerce, logistics management, and related disciplines. Eligibility criteria included basic Chinese reading proficiency, basic web search skills, normal or corrected to normal vision, and the ability to successfully complete eye tracker calibration.

The target sample size was guided by the practical constraints of eye tracking research and the anticipated retention rate of valid eye tracking data. It also reflected the minimum number of participants required for comparisons across the three experimental conditions (Lakens, 2022). Because eye tracking experiments impose relatively stringent requirements regarding the laboratory environment, calibration quality, testing time per participant, and the proportion of valid gaze data, the study was designed to retain at least 30 valid participants in each condition. Additional participants were recruited to account for calibration failure, withdrawal, protocol violations, and valid gaze data rates below 75%. A total of 102 students were recruited. Of the six excluded participants, four had valid gaze data below the prespecified 75% threshold, one could not complete eye-tracker calibration because severe myopia or other eye-related difficulties prevented reliable calibration, and one violated the assigned information-seeking protocol by using AI when it was not permitted. No participant was excluded for withdrawal. Each excluded participant was counted once according to the primary exclusion reason. The final sample comprised 96 participants, with 32 assigned to each of the GenAI-only condition, conventional web search, and hybrid search conditions.

A sensitivity power analysis was conducted to assess whether the final sample adequately supported the primary between group comparisons. For a one way analysis of variance involving three groups, with *α* = 0.05 and statistical power of 0.80, a total sample of *N* = 96 was sufficient to detect an effect of approximately Cohen’s *f* = 0.322, corresponding to *η*^2^ = 0.094. The sample therefore provided adequate power to detect between group effects of moderate magnitude or larger, although its capacity to identify small effects remained limited (Kang, 2021).

#### 3.1.2 Stratification and random assignment

To improve comparability across conditions in terms of educational background and prior experience with AI tools, participants were stratified by year of study, disciplinary cluster, and previous AI use experience. Year of study was classified as first, second, or third year. The disciplinary clusters comprised electromechanical engineering and manufacturing, finance and commerce, logistics management, and related fields. Previous AI use experience was categorized as low, moderate, or high according to scores on the pre experiment questionnaire. Including students from different years and disciplinary backgrounds broadened coverage of learning stages and fields within vocational education. Stratified randomization was used to distribute these characteristics as evenly as possible across conditions and reduce their potential confounding influence on search behavior. The low, moderate, and high AI-experience categories likewise served only as pre-randomization strata and did not constitute additional treatment conditions. Within each stratum, participants were assigned to the three experimental conditions using computer-generated permuted-block randomization. Randomization was performed by a research assistant who was not involved in task scoring, and outcome assessors remained blinded to group allocation during scoring. Permuted-block randomization followed established allocation principles for balancing treatment groups (Lim and In, 2019).

#### 3.1.3 Ethics and informed consent

Ethical approval was obtained from the School of Marxism, Liaoning Economy Vocational and Technical College, before the formal experiment commenced. The approval number was LECVTC-MY-2026-001, and approval was granted on 8 May 2026. The study was conducted in accordance with the ethical principles for research involving human participants set out in the Declaration of Helsinki. Before participation, all students received information about the purpose of the study, the experimental procedures, the types of data to be collected, the anonymization of their data, and their right to withdraw. Written informed consent was obtained from all participants.

### 3.2 Experimental tools and retrieval conditions

To promote comparability, all sessions were conducted in the same laboratory using identical monitors, screen resolutions, browser settings, and network configurations. Task materials, time limits, response requirements, and verification objectives were identical across the three conditions. All participants were instructed to evaluate information, check relevant claims, and revise their answers when necessary. The conditions differed only in the information-access tools available and the resulting affordances for accessing sources.

#### 3.2.1 Standardized environment and GenAI-only condition

Participants in the GenAI-only condition used ChatGPT Plus Instant with the interface language set to Chinese and GPT-5.3 selected as the standardized model. The same test account was used throughout the experiment, with memory disabled or all previous conversations cleared before each session. Access to conventional search engines and external webpages was not permitted. Participants could engage in multi-turn conversations, request explanations or examples, ask follow-up questions, and instruct the system to clarify or reorganize its responses. They were not allowed to use a conventional search engine or independently access external webpages. Source checking in this condition was therefore restricted to citation indicators, source cards, hyperlinks, domain labels, or attribution information displayed within the GenAI interface, when available.

#### 3.2.2 Conventional web-search condition

Participants in the conventional web search condition used Microsoft Edge, version 148.0, and the Bing search engine. The experiment was conducted in mainland China using the local Bing search service. During the data-collection sessions, the mainland-China Bing deployment used in the laboratory did not provide the Copilot Search/generative-answer feature. We additionally reviewed the screen recordings from the conventional web-search condition and confirmed that no AI-generated answer panel, generative summary, or Copilot Search output was displayed on the Bing search-results pages. Participants therefore interacted with standard Bing search results and independently accessed external webpages without exposure to a Bing AI-generated answer layer. Before each session, the browser cache, search history, and login status were cleared to minimize the influence of personalized recommendations on the search results. Participants could independently formulate keyword queries, browse search results, open webpages, compare sources, and synthesize an answer, but they were not permitted to use any generative artificial intelligence tools.

#### 3.2.3 Hybrid-search condition and cross-condition comparability

Participants in the hybrid search condition had access to both the GenAI tool and the search engine described above. The GenAI interface and Bing were opened in separate browser tabs, and participants manually switched between the tabs according to their information-seeking needs; the two interfaces were not displayed side by side. Accordingly, interface transitions and revisits should be interpreted in the context of manual browser-tab switching. They could use either tool as needed to develop a problem framework, generate keywords or an initial response, verify sources, confirm factual claims, and revise their answers. The task materials, time limits, response requirements, and laboratory environment were held constant across all three conditions; the conditions differed only in the forms of information-seeking support available to participants.

### 3.3 Task development and experimental procedure

#### 3.3.1 Task development, expert review, and pilot testing

During the preliminary phase, three vocationally contextualized candidate tasks were developed in accordance with a risk-sensitive principle. The tasks addressed e-commerce after-sales rules, food safety in catering services, and equipment-battery safety; each involved issues of rule applicability, recency, or safety consequences that warranted verification. To reduce potential confounding due to differences in task-material type, four experts in vocational education and educational technology reviewed the candidates, and a pilot test was conducted with 15 students. The selection criteria included task difficulty, vocational relevance, linguistic complexity, verifiability, and completion time. The e-commerce after sales policy task was ultimately selected for the formal experiment. All three conditions completed the same task under the same time limit.

#### 3.3.2 Pretest and eye-tracking calibration

Each experimental session lasted approximately 40 to 60 min. Upon arrival at the laboratory, participants first read and signed the informed consent form. They then completed a pre experiment questionnaire covering demographic information, prior AI use experience, web search experience, acceptance of AI, and baseline trust. A five point eye tracker calibration was subsequently performed.

#### 3.3.3 Information-integration task

During the main task, participants completed a vocationally contextualized information integration task under their assigned information seeking condition, with a time limit of 20 min. The task specified a professional role, a realistic work related scenario, conflicting or incomplete information, and relevant operational constraints. Participants were required to locate and evaluate information, verify pertinent claims, and propose appropriate revisions or solutions. Eye movements and on-screen activity were recorded throughout the task.

#### 3.3.4 Immediate near-transfer task

Immediately afterward, participants completed a 10 min immediate near transfer task under the same assigned information seeking condition. Immediate near transfer was defined as the application of source verification, evidence comparison, and answer revision strategies to a new but structurally similar vocational problem. The transfer task altered the case topic, task objective, pattern of conflicting evidence, and decision constraints, while retaining the same vocational domain and a comparable level of difficulty. It was therefore designed to assess immediate near transfer rather than delayed transfer, cross domain far transfer, or independent transfer without technological support (Forsyth, 2018).

Maintaining the assigned condition during the transfer task made it possible to examine how verification strategies were reapplied within different information-seeking environments. The resulting scores should therefore be interpreted as condition-supported immediate near-transfer performance rather than independent performance without search or GenAI support. Accordingly, this measure was not treated as evidence of long-term “capability development,” but was operationalized as verification-related performance and the immediate application of verification strategies in a structurally similar task.

#### 3.3.5 Post-test and data export

After completing both tasks, participants responded to a post experiment questionnaire. The researchers then exported the eye tracking data, organized the screen recordings, and collected the participants’ written responses. The experimental procedure and multimodal data collection workflow are presented in Figure 2.

**Figure 2:**
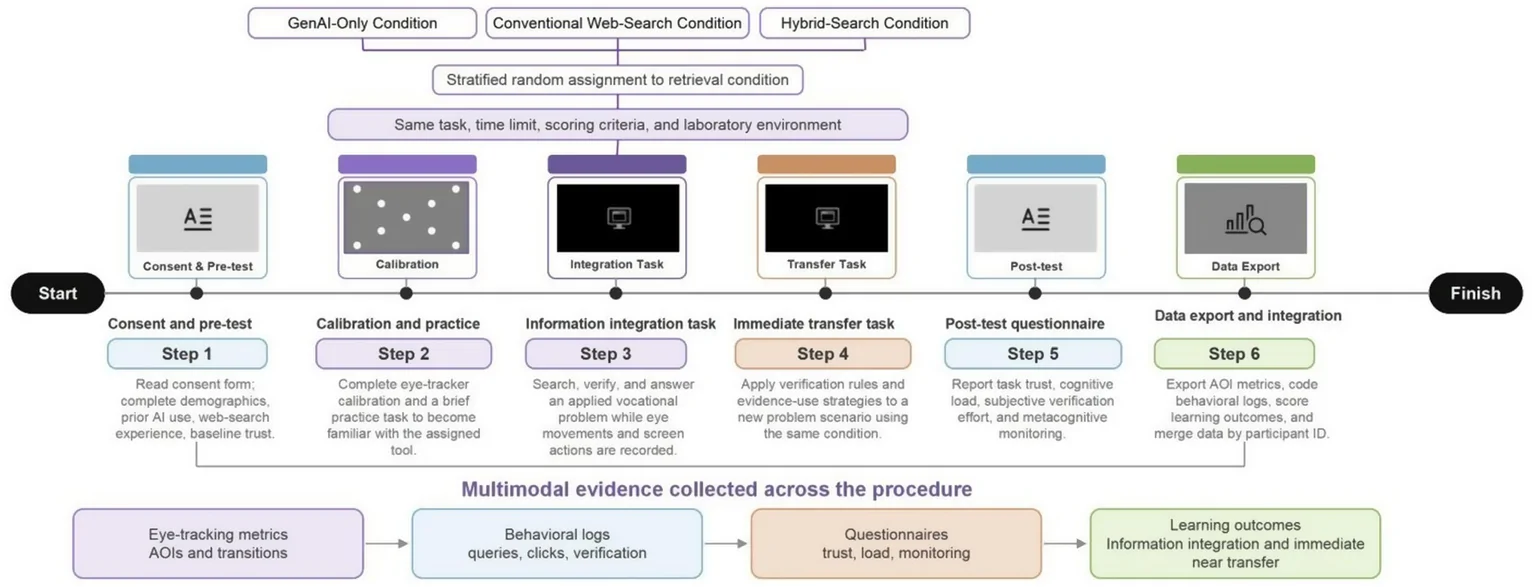
Experimental workflow and multimodal data collection.

### 3.4 Eye tracking apparatus, AOI definition, and multimodal measures

#### 3.4.1 Eye-tracking apparatus and laboratory setup

Eye tracking data were collected using a Tobii Pro Spark screen based eye tracker at a sampling rate of 60 Hz. Tobii Pro Lab 1.232 was used for recording playback, data preprocessing, and area of interest (AOI) annotation. Each participant completed an eye tracker calibration before the formal experiment. All sessions were conducted under stable lighting conditions, with the screen resolution and display scaling held constant (Carter and Luke, 2020; Orquin and Holmqvist, 2018).

#### 3.4.2 Functional AOI framework

Because GenAI interaction pages, search engine results pages, and external webpages differ substantially in their visual structure, AOIs were defined according to functional equivalence rather than fixed spatial coordinates. Four page types were distinguished, the task page, the GenAI interaction page, the search results page, and external source webpages. The task page comprised a task instruction area and a response entry area. The GenAI interaction page included a prompt entry area, a GenAI generated response area, and a source or citation cue area. The conventional web search interface comprised a search results area, a webpage content area, and a source information area.

For cross condition analyses, functionally comparable regions were aggregated into four higher level categories: the task comprehension AOI, information acquisition AOI, source evidence AOI, and answer construction AOI. This classification enabled attention to equivalent information functions to be compared despite differences in page layout and interface structure (Hooge et al., 2026).

#### 3.4.3 Dynamic page states and AOI normalization

The interfaces were scrollable and changed dynamically during interaction (Blascheck et al., 2017). AOIs were therefore coded according to the content visible in each recorded page state rather than treated as permanently fixed screen coordinates. A new page state was marked whenever navigation, scrolling, or newly generated content altered the visible layout. AOI boundaries were updated accordingly, and fixation duration was accumulated across all page states assigned to the same functional AOI category. Content outside the visible viewport was excluded from dwell time calculations. This procedure enabled attention to functionally equivalent information regions to be aggregated across page transitions, scrolling events, and changing interface layouts.

Source cues were defined according to the information displayed within each interface. On the GenAI interface, these cues included visible citation markers, source cards, hyperlinks, publisher or domain labels, and explicit statements regarding the provenance of generated claims. On search results pages, source cues included result titles, URLs or domains, publication dates, publisher or institutional identifiers, and source related information presented in the result snippets. On external webpages, source cues included author, institutional, or publisher information, publication dates, URLs or domains, reference lists, and identifiers associated with official policies, regulations, or documents.

When no source information was displayed in a given page state, no source evidence AOI was coded for that state. The absence of a source evidence AOI was therefore treated as an interface characteristic rather than as evidence that the participant had ignored available source information. The AOI definitions were established before the primary analyses and were applied consistently to all recordings using the same functional coding protocol.

To reduce the influence of variation in the number of pages visited and total browsing time, AOI measures were normalized relative to each participant’s total valid fixation duration or valid viewing time, as appropriate. The proportion of fixation duration within the source evidence AOI was calculated by dividing fixation duration within that AOI by the participant’s total valid fixation duration. Gaze data across pages were aggregated by functionally equivalent AOI category rather than compared on the basis of individual page layouts or fixed screen positions.

#### 3.4.4 Eye-tracking indicators and interpretive boundaries

The principal eye-tracking measures were total fixation duration, fixation count, dwell proportion within each AOI, revisit count, and transitions between AOIs. These indices characterized overall attention allocation, attention to source evidence, repeated checking, and switching among functional regions. By themselves, however, they indicate only visual attention and viewing behavior; they cannot establish that participants understood the evidence or engaged in critical evaluation. They were therefore interpreted jointly with behavioral logs, cross-verification, answer revision, and task performance. A schematic illustration of the AOI definitions is provided in Figure 3.

**Figure 3:**
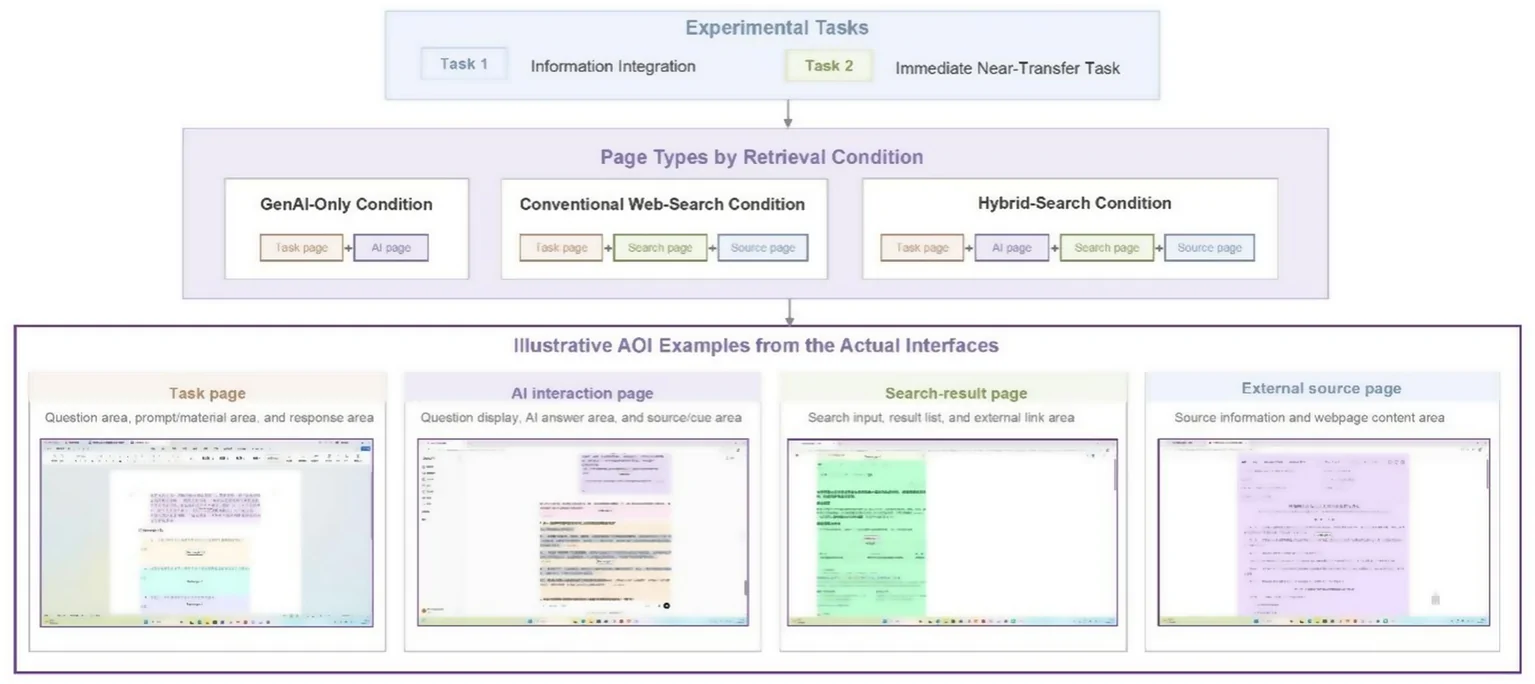
Areas of interest for eye tracking analysis.

#### 3.4.5 Behavioral-log coding and verification definitions

Behavioral logs were derived from continuous screen recordings and manual coding. Coded variables included the number of prompts or queries, page switches, source clicks, external webpages visited, whether cross-verification occurred, total completion time across the two timed tasks, direct copying of AI output, and the number of sources cited in the final response. The total-completion-time measure was the summed active completion time across the 20-min information-integration task and the 10-min immediate near-transfer task, with a maximum possible value of 30 min. Two coders independently coded the recordings using a standardized scheme; disagreements were resolved through discussion, and intercoder reliability was reported. To avoid conflating different levels of behavior under the general label of “verification,” we distinguished source access, a verification attempt, and independent cross-source verification. Source clicks and webpage visits primarily indicated source access. Because participants in the GenAI-only condition could not independently access external webpages, asking GenAI to provide a second source for the same factual claim was coded as a cross-source verification attempt. In the conventional web-search and hybrid-search conditions, independent cross-source verification was coded when a participant actually accessed and compared at least two mutually independent external sources. The proportions reported across all three conditions therefore describe cross-source verification attempts in a broad, affordance-specific sense; their implementations were not equivalent to independent external verification.

#### 3.4.6 Questionnaire measures and learning outcomes

The pre- and post-experiment questionnaires used five-point Likert-type scales to collect demographic information and measure AI acceptance, task trust, overall perceived cognitive load, perceived verification effort, and metacognitive monitoring. Internal consistency was assessed before the primary analyses using Cronbach’s *α* (Taber, 2018). Learning-outcome ratings covered information accuracy, completeness of argumentation, source quality, and quality of transfer application. Cognitive load was measured once in the post-task questionnaire after both tasks and was operationalized as overall perceived cognitive load, reflecting the general mental effort and task-processing burden experienced during the assigned search-and-response workflow. Higher scores indicated greater overall perceived load. The measure did not distinguish intrinsic load, extraneous load, or learning-relevant cognitive investment.

Learning outcomes comprised performance on the information integration task and the immediate near transfer task. Responses were evaluated in terms of information accuracy, completeness of argumentation, source quality, and the quality of transfer application. Each dimension was rated on a five point scale, and the dimension scores were combined to produce a total score. Two raters independently evaluated all responses using a standardized scoring rubric and completed calibration training before formal scoring. Inter rater reliability was calculated after scoring. When reliability met the predefined criterion, the mean of the two ratings was used as the final score. Cases involving substantial disagreement were reviewed by a third rater. The study variables, corresponding indicators, data sources, and their roles in the analyses are summarized in Table 1.

**Table 1:** Variables, indicators, data sources, and analytical roles.

Variable category	Core indicators	Data source	Analytical role
Eye tracking measures	Total fixation duration, fixation count, source-evidence AOI dwell proportion, revisit count, and AOI transition count	Data exported from Tobii Pro Lab	Characterizing attention allocation, attention to source information, and switching between functional regions
Behavioral logs	Prompts/queries, page switching, source clicks, external-webpage visits, cross-verification attempts, copying, answer revision, and total completion time across the two timed tasks	Screen recordings and manual coding	Characterizing search pathways, depth of verification, and reliance on technological tools
Questionnaire measures	Demographic characteristics, AI acceptance, task trust, cognitive load, perceived verification effort, and metacognitive monitoring	Pre and post task questionnaires	Assessing subjective judgments, perceived cognitive demands, and verification tendencies
Learning outcomes	Information integration task quality and immediate near-transfer performance	Task responses and scoring rubric	Evaluating learning outcomes and the immediate application of verification strategies

### 3.5 Data preparation and statistical analysis

#### 3.5.1 Data preprocessing and quality control

Eye tracking data were exported for the predefined areas of interest (AOIs) and subjected to quality screening. Participants were excluded if calibration failed, substantial gaze drift was observed, or the proportion of valid gaze data fell below the predefined threshold of 75%. In the recruited sample, exclusions comprised insufficient valid gaze data below 75% (*n* = 4), eye-tracker calibration failure related to severe myopia or other eye difficulties (*n* = 1), and a protocol violation involving unauthorized AI use (*n* = 1). Behavioral logs were independently coded by two coders, and task responses were independently evaluated by two raters. Intercoder and interrater reliability were calculated separately. Eye tracking measures, behavioral indicators, questionnaire responses, and task performance scores were then merged by participant ID to create the final analytical dataset.

#### 3.5.2 General analytical strategy and assumption checks

Statistical analyses were conducted using IBM SPSS Statistics, version 26.0. Descriptive statistics were calculated for the principal variables, and distributional characteristics and homogeneity of variance were examined before inferential testing. Variables satisfying the assumptions of parametric analysis were analyzed using one way analysis of variance (ANOVA), followed by appropriate *post hoc* comparisons. When these assumptions were not met, the Kruskal-Wallis *H* test was used. Categorical variables, including the occurrence of cross verification, were analyzed using Chi-square tests or Fisher’s exact tests when expected cell frequencies were insufficient. For each inferential test, *α* = 0.05 served as the decision threshold. Values of *p* < 0.05 were treated as sufficient statistical evidence to reject the corresponding null hypothesis, whereas *p* ≥ 0.05 was treated as insufficient evidence to reject it. Results are reported as frequencies and percentages or as *M* ± SD, as appropriate, together with the test statistic, *p*-value, effect size, and confidence interval where applicable. SPSS 26.0 supported the required assumption checks, ANOVA, categorical and nonparametric tests, correlations, hierarchical regression, and diagnostic analyses and served as the principal statistical package. Heatmaps and scanpaths were used only to support descriptive interpretation of visual-search strategies; they were not used as the primary basis for significance testing.

#### 3.5.3 RQ1: visual attention and verification behavior

For RQ1, the analyses examined between-condition differences in source related visual attention and observable verification behavior. The primary visual attention measures were source-evidence AOI dwell proportion, revisits to source-related regions, and transitions between functionally distinct AOIs. Behavioral indicators included requests for supporting evidence, citation use, copying behavior, answer revision, and cross-verification. Because access to external information differed by condition, source clicks and external-webpage visits were reported as condition-specific descriptive measures. The three-condition comparison first characterized overall behavioral differences among information-seeking workflows. A “cross-source verification attempt” in the GenAI-only condition refers to requesting a second source from GenAI for the same claim, whereas in the two web-enabled conditions it could involve independent external cross-source verification. The latter was examined separately in a supplementary comparison restricted to the conventional web-search and hybrid-search conditions.

#### 3.5.4 RQ2: subjective measures and task performance

For RQ2, the three conditions were compared in task trust, overall perceived cognitive load, and immediate near-transfer performance. One way ANOVA or the Kruskal Wallis H test was used as appropriate, with *post hoc* comparisons following a significant omnibus test. To avoid conflating efficiency with verification quality or learning outcomes, operational efficiency was defined exclusively by combined active completion time across the two timed tasks and overall perceived cognitive load. Verification was represented separately by source-evidence AOI dwell, revisits, cross-source verification attempts, and answer revision; task performance was represented by the information-integration and immediate near-transfer scores. Task trust was reported as a separate subjective-judgment variable and was not included in the definition of operational efficiency. Perceived verification effort, metacognitive monitoring, and information-integration performance were reported as secondary outcomes that supplemented the three constructs named in RQ2.

#### 3.5.5 RQ3: correlation, hierarchical regression, and sensitivity analysis

For RQ3, zero-order correlations and hierarchical regression were used to examine whether source related attention and cross-verification were associated with immediate near transfer performance. Search condition, prior AI-use experience, and prior-web search experience were entered before the focal process indicators, allowing their associations with near-transfer performance to be estimated after accounting for condition and prior familiarity with the two information-seeking tools. Multicollinearity among predictors was assessed using tolerance and the variance inflation factor (VIF) to confirm that source clicks, cross-verification, and the other predictors could be included simultaneously in the final model.

Because the conditions did not provide equivalent access to independent external sources, regression findings involving cross-verification were interpreted in light of each condition’s affordances. When independent external cross-source verification was used as a predictor, a sensitivity analysis was conducted within the two web-enabled conditions. Regression coefficients were interpreted as statistical associations rather than evidence of causal effects. In addition, task trust and overall perceived cognitive load were both measured at the same post-task assessment after the two tasks, whereas verification behavior was obtained from process records. Because the design did not establish the temporal ordering required for causal mediation, we did not estimate a causal mediation model with task trust as a mediator. Instead, zero-order correlations and multivariable regression were used to characterize statistical associations among overall perceived cognitive load, task trust, verification behavior, and immediate near-transfer performance.

## 4 Results

### 4.1 Data quality, measurement reliability, and baseline equivalence

#### 4.1.1 Data quality and measurement reliability

The final analysis included 96 participants, with 32 assigned to each of the GenAI only search, conventional web search, and hybrid search conditions. Among the retained participants, the mean proportion of valid gaze data was 88.64%, SD = 5.73%, exceeding the predefined threshold of 75%. Cronbach’s *α* coefficients for the principal questionnaire scales ranged from 0.81 to 0.88, while Cohen’s *κ* values for behavioral coding ranged from 0.82 to 0.91. Inter rater reliability for the information integration and immediate near transfer task scores was ICC = 0.89 and ICC = 0.87, respectively. No severe outliers were identified among the main continuous variables, and their distributional characteristics and homogeneity of variance were generally adequate for the subsequent analyses.

#### 4.1.2 Baseline equivalence across conditions

Baseline balance tests revealed no significant differences among the three conditions in gender, age, prior AI use experience, web search experience, AI acceptance, or baseline task trust, all *p* > 0.05, supporting the broad comparability following stratified randomization. To improve transparency, Table 2 also presents the distributions of year of study, disciplinary cluster, and low/moderate/high prior AI-use strata for the full sample and each condition; these strata did not constitute additional experimental groups. Prior AI-use experience and web-search experience were nevertheless retained as covariates in the regression models to account for individual differences in familiarity with the two information-seeking tools.

**Table 2:** Participant characteristics and baseline equivalence across conditions.

Variable	Total (*N* = 96)	GenAI-only (*n* = 32)	Conventional web search (*n* = 32)	Hybrid search (*n* = 32)	Test statistic	*p*
Total	96	32	32	32		
Gender, male/female	45/51	15/17	14/18	16/16	*χ*^2^ = 0.25	0.883
Age, years	20.39 ± 1.20	20.31 ± 1.18	20.47 ± 1.26	20.38 ± 1.21	*F* = 0.14	0.872
Year of study, *n*					*χ*^2^ = 0.13	0.998
First year	33	11	11	11		
Second year	32	11	10	11		
Third year	31	10	11	10		
Disciplinary cluster, *n*					*χ*^2^ = 0.13	0.998
Electromechanical	33	11	11	11		
Finance/commerce	32	10	11	11		
Logistics/related fields	31	11	10	10		
Prior AI-use experience	3.24 ± 0.76	3.24 ± 0.76	3.18 ± 0.81	3.31 ± 0.74	*F* = 0.22	0.806
Prior AI-use category, *n*					*χ*^2^ = 0.19	0.996
Low	31	10	10	11		
Moderate	34	12	11	11		
High	31	10	11	10		
Web-search experience	3.77 ± 0.68	3.72 ± 0.68	3.81 ± 0.71	3.78 ± 0.66	*F* = 0.13	0.879
AI acceptance, pretest	3.45 ± 0.62	3.46 ± 0.62	3.39 ± 0.65	3.51 ± 0.59	*F* = 0.31	0.734
Baseline task trust	3.27 ± 0.57	3.28 ± 0.57	3.22 ± 0.61	3.30 ± 0.55	*F* = 0.17	0.844

Continuous variables are reported as *M* ± SD.

### 4.2 Source related visual attention and verification behavior

#### 4.2.1 Source-related visual attention

To address RQ1, source-related visual attention and observable verification behavior were compared across the three information-seeking conditions. Total fixation duration did not differ significantly, whereas fixation count, source-evidence AOI dwell proportion, source-related revisits, and cross-AOI transitions did. The main statistical results are presented in Table 3.

**Table 3:** Eye tracking and behavioral measures across the three information access conditions.

Measure	GenAI only	Conventional web search	Hybrid search	Test statistic	*p*	Effect size
Panel A. Eye tracking measures						
Total fixation duration (s)	1,098 ± 118a	1,123 ± 129a	1,110 ± 121a	*F* = 0.33	0.718	ηp^2^ = 0.007
Fixation count	2,850 ± 420b	3,220 ± 460a	3,380 ± 450a	*F* = 12.01	<0.001	ηp^2^ = 0.205
Source evidence AOI dwell proportion (%)	11.8 ± 7.1c	22.7 ± 8.0b	27.9 ± 8.6a	*F* = 34.41	<0.001	ηp^2^ = 0.425
Source related revisit count	3.2 ± 2.1c	7.1 ± 3.0b	9.4 ± 3.3a	*F* = 38.81	<0.001	ηp^2^ = 0.455
AOI transition count	18.6 ± 8.2c	42.4 ± 13.1b	50.7 ± 14.6a	*F* = 58.96	<0.001	ηp^2^ = 0.559
Panel B. Behavioral measures						
Total completion time across the two timed tasks (min)	24.2 ± 3.4c	29.1 ± 4.1a	26.8 ± 3.7b	*F* = 13.72	<0.001	ηp^2^ = 0.228
Source clicks	0.9 ± 0.8b	4.8 ± 1.5a	5.7 ± 1.8a	*F* = 101.95	<0.001	ηp^2^ = 0.687
External webpages visited	Not available	5.5 ± 2.2a	4.9 ± 2.1a	*t* = 1.12	0.269	*d* = 0.28
Cross-verification attempt, *n* (%)	8 (25.0)b	22 (68.8)a	25 (78.1)a	*χ*^2^ = 21.03	<0.001	*V* = 0.468
Direct copying, *n* (%)	14 (43.8)a	0 (0.0)b	4 (12.5)b	*χ*^2^ = 21.33	<0.001	*V* = 0.471
Answer revision, *n* (%)	10 (31.3)b	19 (59.4)ab	25 (78.1)a	*χ*^2^ = 14.48	0.001	*V* = 0.388
Sources cited in final response	1.3 ± 1.0b	3.4 ± 1.4a	4.1 ± 1.5a	*F* = 39.12	<0.001	ηp^2^ = 0.457

Continuous variables are shown as *M* ± SD and categorical variables as *n* (%). Within each row, values that do not share a letter differ at the multiplicity adjusted *p* < 0.05 level. Source clicks and external webpage visits are condition dependent affordance indicators, the external webpage comparison was restricted to the two web enabled conditions.

The clearest pattern concerned attention to source evidence: source-evidence dwell and related revisits and transitions were lowest in the GenAI-only condition and highest in the hybrid-search condition. GenAI-only participants concentrated more attention within the information-acquisition region, whereas hybrid-search participants shifted more often between content, source evidence, and response construction. Figure 4 provides the corresponding AOI distribution.

**Figure 4:**
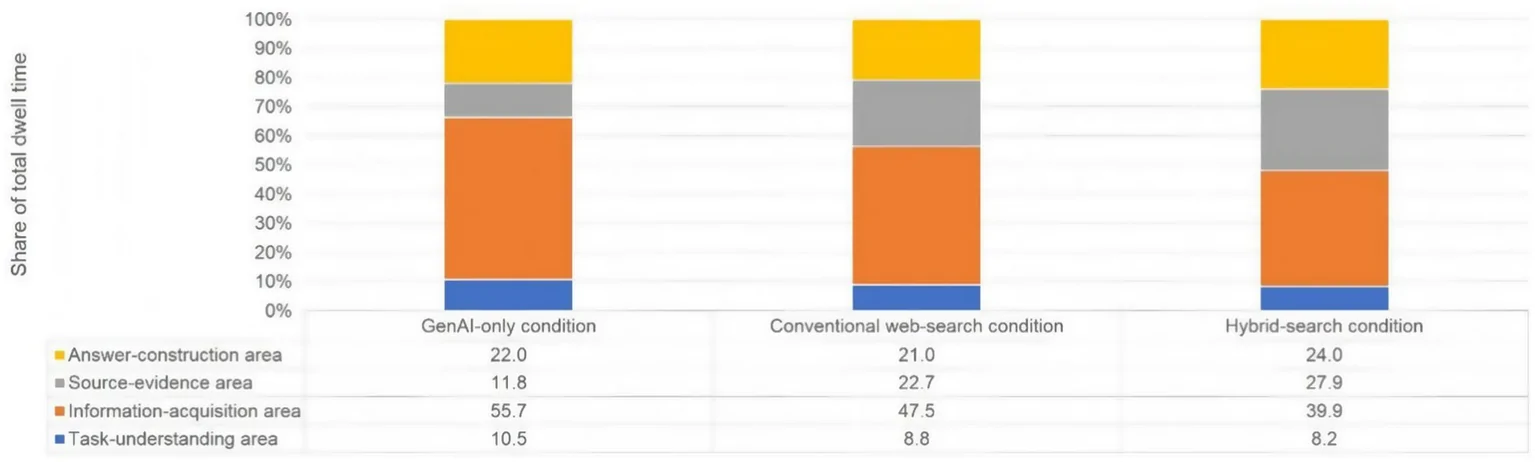
Attention allocation across four functional AOIs.

#### 4.2.2 Observable verification behavior

Behavioral logs showed the same overall contrast (Table 3). GenAI-only participants had the shortest combined active completion time across the two timed tasks but showed less cross-verification and answer revision and more direct copying, whereas hybrid search maintained relatively high verification and revision with less time than conventional web search. Because external-source access differed by condition, source clicks and webpage visits are best interpreted as affordance-dependent measures rather than pure indicators of verification motivation. In the GenAI-only condition, the 25.0% cross-verification-attempt rate specifically denotes requests to GenAI for a second source for the same claim; independent external comparison was possible only in the web-enabled conditions.

The exploratory temporal plot was consistent with this pattern: GenAI-only participants moved quickly to the AI interface, conventional web-search participants progressed toward external webpages, and hybrid-search participants alternated more often among the AI interface, search results, and source webpages (Figure 5).

**Figure 5:**
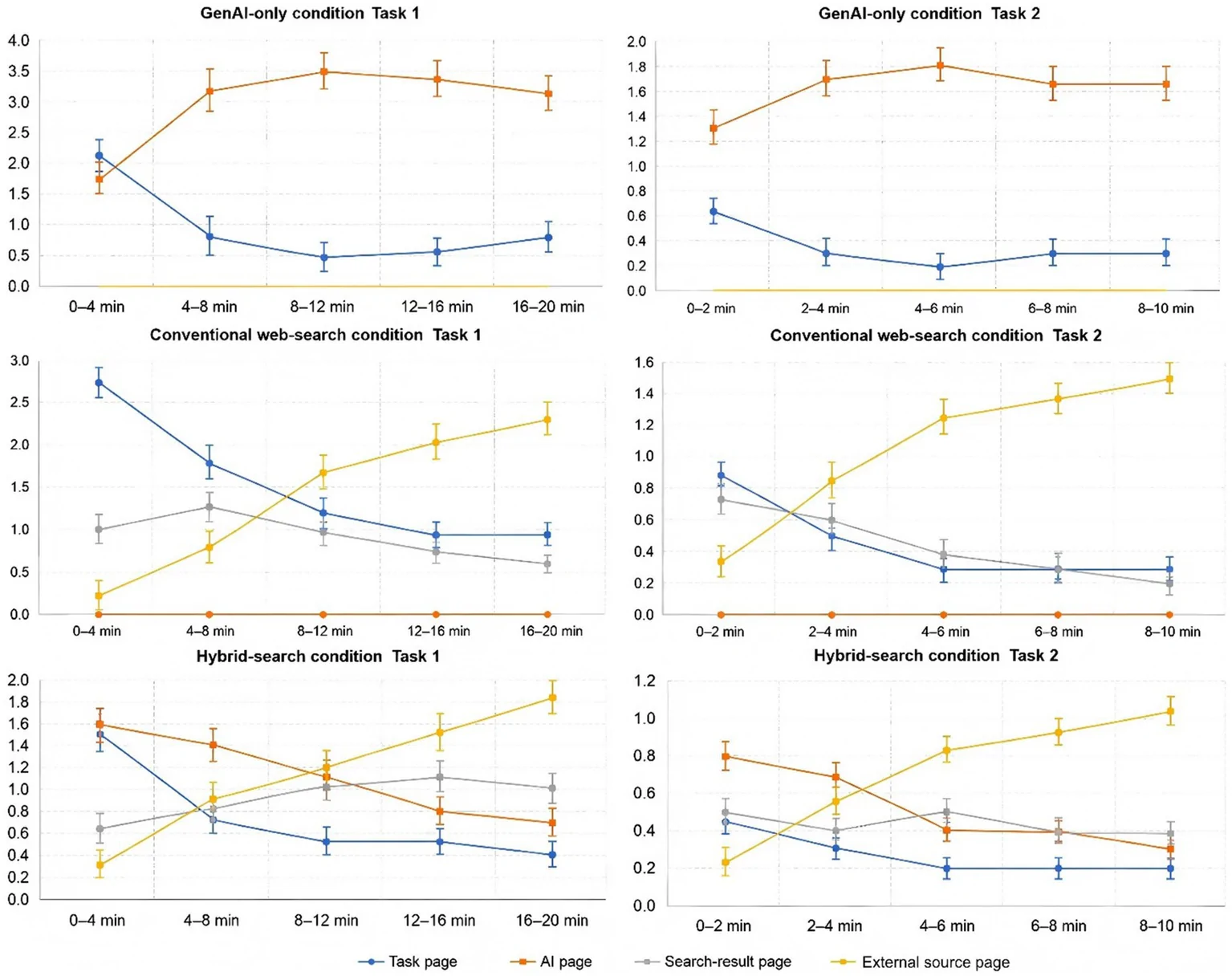
Exploratory time course visualization of page dwell time across tasks and information access conditions. Error bars represent the standard error of the mean.

### 4.3 Questionnaire measures and task performance

#### 4.3.1 Overall group differences and pairwise comparisons

To address RQ2, the three conditions were compared in terms of task trust, overall perceived cognitive load, perceived verification effort, metacognitive monitoring, information-integration performance, and immediate near-transfer performance. Omnibus tests were significant for all six outcomes; complete descriptive statistics, effect sizes, and adjusted pairwise comparisons are reported in Table 4.

**Table 4:** Questionnaire measures and task performance across the three information access conditions.

Variable	GenAI only	Conventional web search	Hybrid search	*F* (2, 93)	*p*	ηp^2^
	*M* ± SD					
Task trust	4.18 ± 0.49a	3.42 ± 0.58c	3.74 ± 0.53b	16.3	<0.001	0.26
Cognitive load	3.21 ± 0.57b	3.91 ± 0.55a	3.48 ± 0.52b	13.33	<0.001	0.223
Perceived verification effort	3.12 ± 0.62b	3.76 ± 0.54a	4.01 ± 0.50a	21.84	<0.001	0.32
Metacognitive monitoring	3.35 ± 0.56c	3.71 ± 0.52b	4.02 ± 0.47a	13.41	<0.001	0.224
Information integration task score	78.34 ± 7.16b	80.72 ± 7.88ab	84.41 ± 6.92a	5.57	0.005	0.107
Immediate near transfer task score	76.99 ± 8.25b	81.65 ± 8.20b	86.99 ± 7.45a	12.61	<0.001	0.213

Questionnaire variables were measured on 5-point Likert type scales, task scores were transformed to a 100 point scale. Means within a row that do not share a letter differ significantly in Tukey HSD comparisons at *p* < 0.05.

The key contrasts were as follows. GenAI-only search produced the highest task trust and the lowest overall perceived cognitive load, whereas hybrid search produced the highest metacognitive monitoring and the strongest immediate near-transfer performance. Perceived verification effort was highest under hybrid search but did not differ significantly from conventional web search. Hybrid search significantly outperformed GenAI-only search on both information-integration and near-transfer performance and also outperformed conventional web search on near-transfer performance. Overall perceived cognitive load did not differ significantly between the GenAI-only and hybrid conditions.

#### 4.3.2 The high-trust-low-verification pattern

The reliability-relevant process indicators revealed a high-trust–low-verification pattern in the GenAI-only condition. Although task trust was highest, source-evidence attention, cross-verification, and answer revision were lower and direct copying was higher than in the hybrid-search condition (Tables 3 and 4). In hybrid search, trust was lower but verification and revision were substantially more frequent. The 25.0% GenAI-only cross-verification-attempt rate denotes requests for a second AI-provided source rather than independent external-source verification. Taken together, high subjective trust did not ensure substantive evidence processing.

### 4.4 Correlations and hierarchical regression

#### 4.4.1 Zero-order correlations

Zero-order correlations showed that immediate near-transfer performance was positively related to source-evidence AOI dwell proportion (*r* = 0.46, *p* < 0.001) and cross-verification attempts (*r* = 0.52, *p* < 0.001), and negatively related to overall perceived cognitive load (*r* = −0.31, *p* = 0.002). Task trust was not significantly related to near-transfer performance (*r* = −0.14, *p* = 0.172), but it was negatively related to cross-verification attempts (*r* = −0.38, *p* < 0.001), as reported in Table 5. Because source-access measures were shaped by condition-specific affordances, these correlations are descriptive; the hierarchical regression provides the primary adjusted estimates. The analyses concern observable verification behavior rather than a separately measured self-report construct of need for source verification.

**Table 5:** Descriptive statistics and zero-order correlations among process measures and immediate near-transfer performance.

Variable	*M*	SD	1	2	3	4	5	6	7
1. Trust	3.78	0.61	1						
2. Source dwell	20.8	10.9	−0.29**	1					
3. Source clicks	3.8	2.5	−0.34**	0.42**	1				
4. Cross-verification attempt	0.573	0.497	−0.38**	0.48**	0.56**	1			
5. Cognitive load	3.53	0.61	−0.22*	0.18	0.25*	0.12	1		
6. Metacognition	3.69	0.58	−0.30**	0.41**	0.36**	0.45**	−0.26*	1	
7. Near transfer	81.88	8.9	−0.14	0.46**	0.39**	0.52**	−0.31**	0.44**	1

*p < 0.05; **p < 0.01; Trust, task trust; Source dwell, source evidence AOI dwell proportion; Metacognition, metacognitive monitoring; Near transfer, immediate near transfer score. Cross verification was coded 0/1, its correlations with continuous variables are point biserial correlations.

#### 4.4.2 Hierarchical regression

Table 6 presents the hierarchical regression results. The final model explained 51% of the variance in immediate near-transfer performance (adjusted *R*^2^ = 0.46). After adjustment for search condition and prior tool experience, hybrid search (*β* = 0.24, 95% CI [0.04, 0.44], *p* = 0.019), source-evidence AOI dwell proportion (*β* = 0.28, 95% CI [0.08, 0.48], *p* = 0.006), and cross-verification attempts (*β* = 0.31, 95% CI [0.12, 0.50], *p* = 0.002) were positively associated with near-transfer performance, whereas overall perceived cognitive load was negatively associated with performance (*β* = −0.20, 95% CI [−0.38, −0.02], *p* = 0.031). Source clicks were not independently associated with performance once the more substantive verification indicator was included.

**Table 6:** Hierarchical regression predicting immediate near-transfer performance.

Predictor	Model 1*β*	Model 2*β*	Model 3*β*	Approx. 95% CI for *β*	*p*
Prior AI-use experience	0.13	0.08	0.04	[−0.12, 0.20]	0.624
Web-search experience	0.19	0.11	0.06	[−0.10, 0.22]	0.462
Conventional web search		0.21*	0.09	[−0.10, 0.28]	0.352
Hybrid search		0.43***	0.24*	[0.04, 0.44]	0.019
Task trust			−0.12	[−0.30, 0.06]	0.178
Source-evidence AOI dwell proportion			0.28**	[0.08, 0.48]	0.006
Source clicks			0.13	[−0.04, 0.30]	0.141
Cross-verification attempt			0.31**	[0.12, 0.50]	0.002
Overall perceived cognitive load			−0.20*	[−0.38, −0.02]	0.031
*R* ^2^	0.07	0.26	0.51		
Adjusted *R*^2^	0.05	0.23	0.46		
Δ*R*^2^	0.07	0.19***	0.25***		
*F*	3.51*	7.99***	9.95***		

The GenAI-only condition was the reference category. *p < 0.05; **p < 0.01; ***p < 0.001.

#### 4.4.3 Web-enabled sensitivity analysis

In the web-enabled sensitivity analysis (*n* = 64), independent cross-source verification remained positively associated with immediate near-transfer performance (*β* = 0.29, 95% CI [0.05, 0.53], *p* = 0.021), whereas source clicks were not significant (*β* = 0.12, 95% CI [−0.12, 0.36], *p* = 0.318). This result reduces, but does not eliminate, the interpretive problem created by unequal source-access affordances across all three conditions.

### 4.5 Descriptive eye tracking visualizations

To illustrate the visual search patterns under the three information-seeking conditions, one participant was selected from each condition whose primary eye tracking measures were close to the central tendency of the corresponding group. Heatmaps and scanpath visualizations were then generated for these selected cases. Participant selection was based jointly on the proportion of dwell time within the source-evidence AOI, the number of revisits to source-related regions, and the number of transitions between AOIs, thereby reducing the influence of atypical cases on the visual interpretation.

#### 4.5.1 Heatmap patterns across conditions

The representative heatmaps showed distinct patterns of visual attention on the task page across the three information-seeking conditions. In the conventional web-search condition, fixations were concentrated mainly in the upper portion of the task-material area, whereas attention to the task-requirement area was relatively limited and dispersed. In the GenAI-only condition, fixations were more strongly concentrated within the task-requirement area, indicating a more localized pattern of attention on the task page. By contrast, the hybrid-search condition showed broader visual coverage across the task-material area while retaining a distinct concentration of fixations within the task-requirement area. Overall, the representative heatmaps suggest more localized task-page attention in the GenAI-only and conventional web-search conditions and broader visual coverage in the hybrid-search condition (Figure 6).

**Figure 6:**
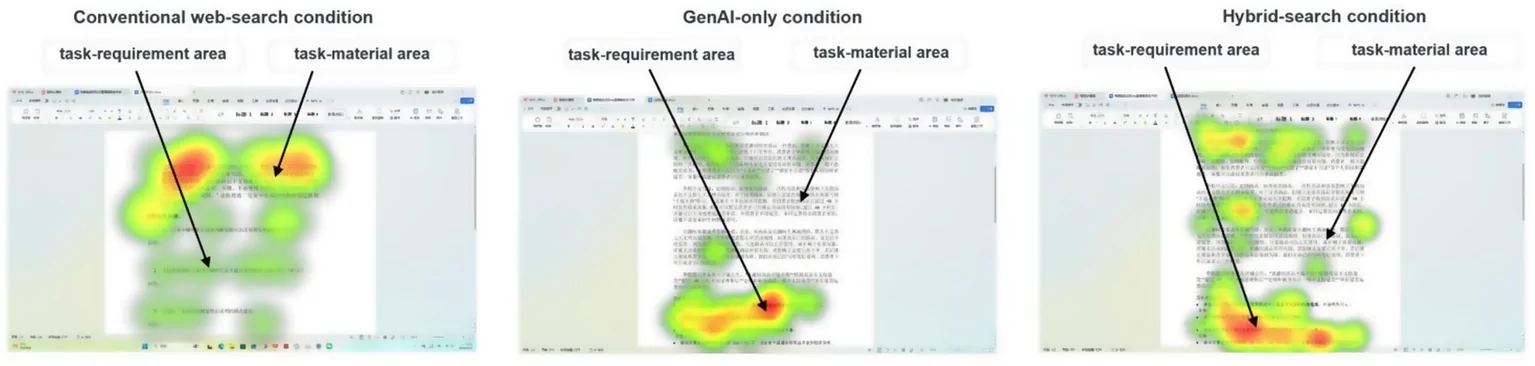
Heatmaps of representative participants across the three search conditions.

#### 4.5.2 Scanpath patterns across conditions

The scanpath further indicated that gaze in the GenAI-only condition remained more often within a single content region, with relatively few transitions among functional regions. The conventional web-search condition showed more switching between search results and webpage content, whereas the hybrid-search condition showed more frequent cross-region revisits, particularly among the generated response, source information, and webpage content. These descriptive visualizations were consistent in direction with the group-level eye-tracking and behavioral results, but they were used only to illustrate possible information-processing pathways, as shown in Figure 7.

**Figure 7:**
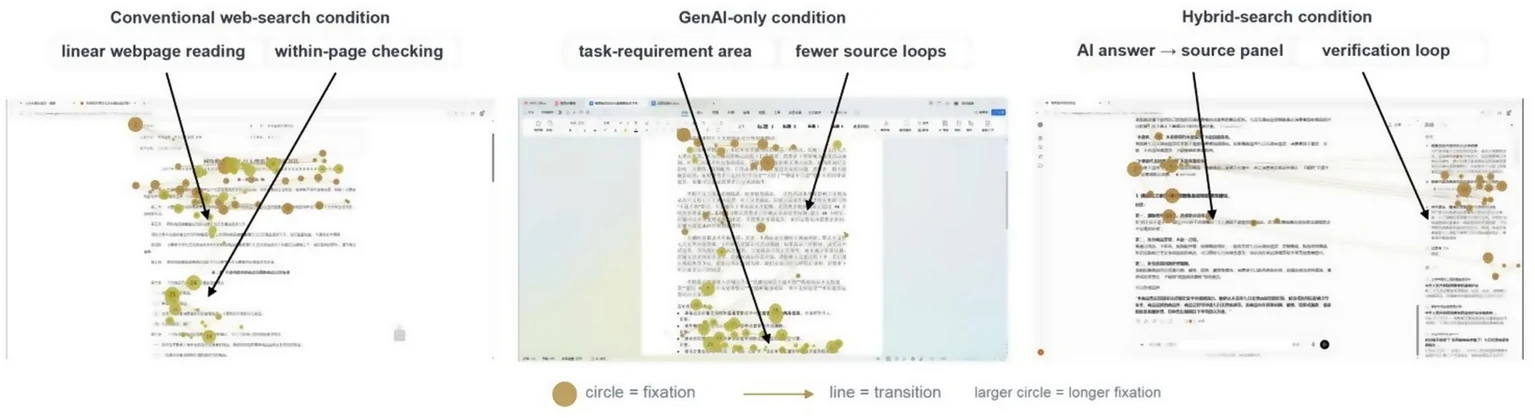
Representative scanpaths across the three search conditions.

## 5 Discussion

The findings indicate that the three information-seeking conditions involved trade-offs among operational efficiency, evidence processing, trust, and task performance rather than a single mode that was superior on every outcome. GenAI-only search was the fastest and produced the lowest overall perceived cognitive load. Conventional web search was the most demanding, but it preserved more extensive multi-source browsing. Hybrid search had intermediate operational costs and was associated with greater source attention, more answer revision, and better immediate near-transfer performance. Task trust likewise did not align uniformly with performance. It was highest in the GenAI-only condition, which had the lowest near-transfer score, lowest in the conventional web-search condition, and intermediate in the hybrid-search condition. Responsible use in vocational education should be evaluated jointly in terms of operational cost, evidence processing, trust calibration, and task performance.

### 5.1 The efficiency–verification trade-off

GenAI reduced the operational effort associated with iterative searching or query reformulation, result screening, multi-page navigation, and information organization, but may also have compressed source tracing, evidence comparison, and judgment of applicability. Eye-tracking and behavioral results showed less source-evidence AOI dwell, fewer source revisits and cross-region transitions, and lower rates of cross-verification and answer revision in the GenAI-only condition. Thus, lower overall perceived cognitive load cannot be equated with higher-quality learning. For combined active completion time across the two timed tasks, hybrid search required only 2.6 min more than GenAI-only search (26.8 vs. 24.2 min), and the difference in overall perceived cognitive load was not significant (3.48 vs. 3.21). Yet source-evidence dwell proportion increased from 11.8 to 27.9%, cross-verification attempts from 25.0 to 78.1%, and answer revision from 31.3 to 78.1%, while direct copying fell from 43.8 to 12.5%. Relative to conventional web search, hybrid search saved 2.3 min in combined active completion time across the two timed tasks, produced lower overall perceived cognitive load (3.48 vs. 3.91), and produced a higher source-evidence dwell proportion (27.9% vs. 22.7%). Its advantage was not maximal efficiency, but a more favorable verification–performance trade-off at moderate operational cost.

From a dual-process perspective, these differences are better understood as differences in how effort was allocated than as a simple contrast between high and low cognitive load. The rapid and coherent output of GenAI may encourage heuristic acceptance with relatively little cognitive effort, whereas source access, explicit verification requirements, and comparison across tools can introduce limited analytic processing at critical evidence checkpoints. Buçinca et al. (2021) similarly found that cognitive forcing functions reduced uncritical reliance on AI advice while increasing perceived effort. The educational objective should not be to maximize cognitive load, but to direct limited cognitive resources toward source comparison, evidential judgment, and answer revision.

This interpretation is consistent with Stadler et al. (2024), who reported that LLMs can reduce the burden of information seeking even when lower cognitive effort is accompanied by shallower reasoning. The higher load associated with conventional web search may include both operational inefficiency and productive effort devoted to evidence evaluation. Hybrid search instead enables a division of labor in which AI supports problem framing and preliminary organization while web search is used to verify key facts and conditions of applicability. Verification can thus be preserved without restoring all the operational costs of conventional search. Rather than eliminating friction, educational design should retain limited, purposeful cognitive friction at critical evidence checkpoints so that AI output remains provisional information subject to verification.

The study measured overall perceived cognitive load and did not distinguish intrinsic load, extraneous load, or learning-relevant cognitive investment. These results do not establish that hybrid search incurred no additional cognitive cost, nor can overall perceived load be treated as a direct indicator of deep processing. Recent review evidence similarly suggests that the effects of GenAI on cognitive load depend on scaffolding, intensity of use, prior knowledge, and task design (Qian et al., 2026). In the present sample, overall perceived cognitive load was not linearly associated with attention to source evidence or cross-source verification attempts. A more plausible interpretation is that some cognitive resources were reallocated from search operations to source verification, evidence comparison, and answer revision. Moreover, the formal task explicitly required participants in all conditions to locate, evaluate, and verify information, while source-access channels differed by condition. Accordingly, the higher verification rate in the hybrid-search condition reflects the combined influence of the verification requirement and tool affordances and should not be equated with a spontaneous verification tendency in naturalistic use.

### 5.2 Trust calibration, verification, and transfer

Task trust did not produce a linear benefit in this study. Trust was highest in the GenAI-only condition (4.18 ± 0.49), whereas immediate near-transfer performance was lowest (76.99 ± 8.25). In the hybrid-search condition, trust was lower (3.74 ± 0.53) and near-transfer performance was highest (86.99 ± 7.45). At the individual level, task trust was not significantly correlated with near-transfer performance (*r* = −0.14, *p* = 0.172). By contrast, source-evidence AOI dwell proportion and the affordance-specific cross-verification-attempt variable remained positively associated with near-transfer after adjustment for condition and prior experience. Reliable use depends not on maximizing trust, but on adjusting trust to the sufficiency and results of verification. The risk of AI-generated “slop” lies not in its AI-generated status alone, but in the mismatch between trust and evidential quality when low-quality content is presented fluently and without adequate sourcing.

Lee et al. (2025) found that greater confidence in GenAI among knowledge workers was associated with less critical thinking and that, in GenAI-supported work, critical thinking shifted toward information verification, response integration, and task management. The current results extend that finding through eye tracking, behavioral logs, and task scores. Source-evidence AOI dwell proportion, revisits, and transitions captured attention to evidence and movement among functional regions, while cross-verification and answer revision indicated whether judgments changed in response to evidence. These measures, however, cannot independently establish critical evaluation or deep understanding.

The process measures also distinguished source access from source verification. Source clicks no longer explained unique variance after more substantive indicators were included, whereas attention to source evidence and cross-verification attempts remained associated with near-transfer performance. Opening a webpage does not establish that its evidence was understood or used. For this reason, we distinguished source access, cross-source verification attempts, and independent external cross-source verification. In the GenAI-only condition, the 25.0% verification rate represented requests for a second AI-provided source concerning the same claim; independent external verification was possible only in the web-enabled conditions. The value of hybrid search lay in enabling learners to move through a sequence of generating, tracing, comparing, and revising information and to repeat that sequence in a structurally similar task. Its advantage lay primarily in the verification process and immediate performance, not operational efficiency or overall trust. AI literacy should extend beyond tool operation to source sensitivity, evidential judgment, metacognitive monitoring, and dynamic trust calibration (Long and Magerko, 2020; Lee and See, 2004).

### 5.3 Theoretical, design, and educational implications

Theoretically, the findings identify an important boundary condition for a dual-process account of AI reliance. Lower overall perceived cognitive load does not automatically indicate a better learning process, just as greater load does not necessarily indicate deeper processing. What matters is whether uncertainty or high-consequence claims trigger analytic engagement with evidence. The absence of a stable positive association between task trust and immediate near-transfer performance likewise suggests that responsible human-AI collaboration should prioritize trust calibrated to the available evidence rather than simply increasing trust in AI.

These findings frame responsible use as a process that can be observed and shaped through design: AI can support generation and organization, while learners retain responsibility for verification, revision, and final judgment. This division of labor is consistent with human-centered AI principles emphasizing human control, system reliability, and retained accountability (Amershi et al., 2019; Shneiderman, 2020). In vocational education, verification should be risk sensitive. Regulatory, safety-related, procedural, time-sensitive, and other high-consequence information should enter a more stringent verification workflow, whereas low-risk, open-ended, or creative tasks may be supported through lighter prompts or selective checks (OECD, 2026; Miao and Holmes, 2023).

Systems can organize information around explicit claim–evidence relationships. Traceable sources can be placed near factual, policy-related, and quantitative claims and can identify the issuing organization, publication date, location in the original material, and scope of the evidence. AI responses and web-based evidence can be presented side by side, and the system can record source inspection, evidential judgment, and answer revision to create a traceable evidence chain. Source cues alone, however, do not demonstrate that verification has occurred. If citations serve primarily as signals of credibility, learners may infer that cited content is necessarily reliable. Hybrid-search designs should combine uncertainty cues, verification of key claims, comparison of independent sources, and revision in response to new evidence, concentrating additional effort at high-risk or high-uncertainty points.

In instruction, GenAI output can be framed as a provisional proposal rather than a model answer. Tasks involving conflicting sources, changing rules, contextual constraints, or evidential gaps can require students to identify provenance, specify conditions of applicability, and justify whether a recommendation should be accepted. A pre-submission verification prompt can ask students to flag unverified claims and document how their answer changed in response to evidence. Bastani et al. (2025) showed that unconstrained GenAI support could improve performance while the tool was available yet undermine subsequent independent performance, whereas learning safeguards mitigated this effect. Assessment should likewise extend beyond the final product to the evidence-processing pathway. Criteria can include source quality, claim–evidence alignment, cross-source checking, recognition of conflicts, justification of revisions, and communication of uncertainty. In this way, GenAI use can move from maximizing efficiency toward a balanced allocation of tool support and learner responsibility for judgment.

### 5.4 Recommendations and implementation priorities

At the immediate course and assessment level, instructors should treat GenAI output as provisional rather than authoritative. For high-consequence factual, regulatory, numerical, or procedural claims, assignments can require a brief claim–evidence log, verification against an authoritative and current source, and a note explaining how the evidence changed the final answer. Verification prompts should be placed at consequential evidence checkpoints rather than applied indiscriminately to every sentence. Grading criteria should reward source quality, claim–evidence alignment, conflict recognition, justified revision, and communication of uncertainty.

At the medium-term curriculum and system level, vocational institutions should embed risk-sensitive source verification, trust calibration, and documentation of AI-supported decisions within digital-literacy modules and program assessments. Curriculum leaders and teacher-development teams can prepare discipline-specific verification cases, while system and interface designers can provide traceable provenance, publication dates, evidence scope, uncertainty cues, and side-by-side comparison of generated claims with primary sources. Institutions should evaluate these practices through repeated tasks, delayed assessments, and tool-withdrawal checks before treating short-term assisted performance as durable capability development.

### 5.5 Limitations and future research

Several limitations define the scope of the findings. First, the sample came from a single vocational college (*N* = 96). Although the experimental task approximated a vocational context, it simplified extended learning and authentic workplace demands and included an explicit verification requirement that may have elevated verification behavior above everyday GenAI use. Because the task was rule oriented and carried plausible real-world consequences, the value of verification observed here should not be generalized directly to low-risk, open-ended, or creative tasks. Second, external-source affordances differed across the three conditions, meaning that differences in cross-verification reflect both behavioral choice and tool availability. The sensitivity analysis within the two web-enabled conditions reduced this confounding but could not decompose the complete workflow effect into pure tool and motivational components. The study also did not directly manipulate AI-generated “slop,” model hallucination rates, or source traceability. It therefore provides evidence about verification behavior and trust calibration, not a direct test of learners’ ability to identify low-quality generated content.

Third, the immediate near-transfer task retained the assigned support condition and therefore measured tool-supported transfer rather than independent performance after tool withdrawal. Without a tool-withdrawal test, delayed follow-up, or repeated measurement, the findings cannot support claims about long-term capability development. Eye tracking primarily measures visual attention, while screen recordings and logs capture observable behavior; neither can independently establish comprehension, deep processing, or critical evaluation. Interpretation therefore relied on converging evidence from gaze, cross-verification, answer revision, and task scores. Task trust and overall perceived cognitive load were also measured at the same post-task assessment, precluding the temporal ordering required for mediation analysis. Accordingly, the study did not test a causal pathway from cognitive load through task trust to source verification. Finally, behavioral intention, effort expectancy, facilitating conditions, and other adoption factors were not measured. The study therefore evaluates execution under assigned conditions rather than the mechanisms governing spontaneous adoption in natural use (BinJwair, 2025). The study also did not measure self-efficacy, need for cognition, epistemic vigilance, or habitual dependence on a particular information-seeking mode; these unmeasured individual differences may influence both tool choice and verification behavior.

Future research should include a wider range of institutions, disciplines, tasks, and AI tools and incorporate delayed and tool-withdrawal assessments. It should also measure overall perceived cognitive load, trust, and subsequent verification dynamically over time. Experimental work should also manipulate verification-prompt intensity, task risk, generated-content quality, and source traceability to examine spontaneous adoption of hybrid strategies, retention of verification behavior, and trust calibration. Combining eye tracking with interviews and classroom-process data may further clarify the mechanisms involved. Larger prospectively designed studies should additionally model self-efficacy, need for cognition, epistemic vigilance, and habitual tool dependence as potential moderators of verification and transfer. Where mediation is theoretically proposed, future studies should establish temporal ordering through repeated or experimentally separated measurements before estimating indirect effects.

## 6 Conclusion

This study compared three information-seeking workflows. GenAI-only search, conventional web search, and hybrid search. The results revealed trade-offs among operational efficiency, evidence processing, and task performance. GenAI-only search was the fastest and produced the lowest overall perceived cognitive load, but it was accompanied by less attention to source evidence, fewer cross-source verification attempts, and fewer answer revisions. Conventional web search required the most time and produced the greatest perceived load, while preserving more multi-source browsing and evidence comparison. Hybrid search had intermediate operational costs and produced greater source-related attention, more answer revision, and better condition-supported immediate near-transfer performance. It is more appropriately described as producing a favorable verification–performance profile under the present experimental conditions, rather than as the most efficient method. Because source-access affordances differed across conditions, the observed differences reflect the combined effects of complete workflows and tool availability and cannot be attributed solely to verification motivation.

Task trust showed no stable positive relationship with immediate near-transfer performance. By contrast, the proportion of dwell time within the source-evidence AOI and the affordance-specific cross-verification-attempt variable remained significantly associated with near-transfer after controlling for search condition and prior tool experience. Source clicks showed no independent association. The same pattern appeared in the sensitivity analysis restricted to the two web-enabled conditions. These results sharpen the distinction between source access and source evaluation: opening a source is not equivalent to verifying it. What appears more transferable is comparing evidence concerning the same factual claim across sources and revising one’s judgment accordingly. From a dual-process perspective, the more informative mechanism is whether analytic processing is triggered at critical evidence checkpoints, not whether overall trust or overall perceived cognitive load is simply maximized or minimized.

For vocational education, these findings support a conditional model of responsible GenAI use. When verification requirements are activated and tool affordances permit, AI can support problem framing and information organization while learners retain responsibility for source checking, evidential judgment, and final decisions. Instructional and system design should reduce barriers to initiating verification through traceable sources, prompts to check critical claims, and aligned assessment requirements. Verification intensity should be calibrated to task risk rather than imposed uniformly across all tasks.

The conclusions are bounded by the single-institution sample, short-duration tasks, explicit verification requirement, and unequal source-access affordances. Because the immediate near-transfer task retained the assigned tools, the findings should not be generalized to delayed transfer, far transfer, unsupported performance, or long-term capability development. Future research should test the relationships among verification strategies, trust calibration, and learning transfer across institutions, vocational domains, and interface designs, including delayed and tool-withdrawal transfer tasks.

## Data Availability

The original contributions presented in the study are included in the article/supplementary material, further inquiries can be directed to the corresponding author.
